# Family Cluster of Middle East Respiratory Syndrome Coronavirus Infections, Tunisia, 2013

**DOI:** 10.3201/eid2009.140378

**Published:** 2014-09

**Authors:** Fekri Abroug, Amine Slim, Lamia Ouanes-Besbes, Mohamed-Ali Hadj Kacem, Fahmi Dachraoui, Islem Ouanes, Xiaoyan Lu, Ying Tao, Clinton Paden, Hayat Caidi, Congrong Miao, Mohammed Mohammed Al-Hajri, Mokhtar Zorraga, Wissem Ghaouar, Afif BenSalah, Susan I. Gerber, World Health Organization Global Outbreak Alert and Response Network Middle East Respiratory Syndrome Coronavirus International Investigation Team

**Affiliations:** Centre Hospitalier-Universitaire Fattouma Bourguiba, Monastir, Tunisia (F. Abroug, L. Ouanes-Besbes, F. Dachraoui, I. Ouanes);; Centre Hospitalier-Universitaire Charles Nicolle, Tunis, Tunisia (A. Slim, M.-A. Hadj Kacem);; Centers for Disease Control and Prevention, Atlanta, Georgia, USA (X. Lu, Y. Tao, C. Paden, H. Caidi, C. Miao, S.I. Gerber);; Supreme Council of Health, State of Qatar (M. Mohammed Al-Hajri);; Direction des Soins de Santé de Base, Tunis (M. Zorraga, W. Ghaouar, A. BenSalah)

**Keywords:** MERS coronavirus, ARDS, infection, ICU, viruses, Tunisia, MERS-CoV, Middle East respiratory syndrome, family cluster

## Abstract

In 2013 in Tunisia, 3 persons in 1 family were infected with Middle East respiratory syndrome coronavirus (MERS-CoV). The index case-patient’s respiratory tract samples were negative for MERS-CoV by reverse transcription PCR, but diagnosis was retrospectively confirmed by PCR of serum. Sequences clustered with those from Saudi Arabia and United Arab Emirates.

As of May 23, 2014, a total of 635 laboratory-confirmed human cases of Middle East respiratory syndrome coronavirus (MERS-CoV) infections had been reported to the World Health Organization; the epidemic has subsequently accelerated (*1*). Of these patients, 193 (30%) died. This new virus causes disease similar to that caused by severe acute respiratory syndrome coronavirus, but MERS-CoV is genetically distinct (*2*). We investigated a cluster of 3 MERS-CoV cases in 1 family in Tunisia.

## The Cases

**Figure 1:**
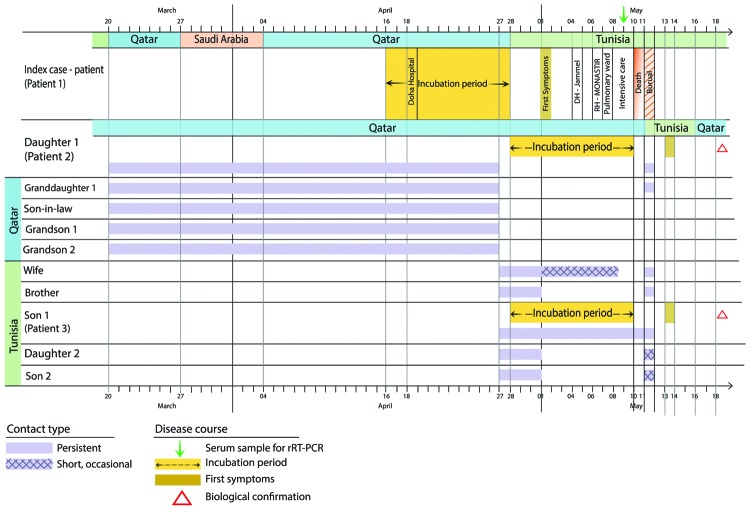
Clinical course of disease for patients with confirmed Middle East respiratory syndrome coronavirus Infection, Tunisia, 2013. RH, regional hospital; DH, district hospital; rRT-PCR, real-time reverse transcription PCR.

 Patient 1, the index case-patient, was a 66-year-old Tunisian man with a 4-year history of untreated diabetes mellitus. During March 20–April 27, 2013, he visited his daughter (patient 2) in Qatar for 5 weeks (Figure 1), 1 week of which they spent on a Muslim pilgrimage to Mecca, Kingdom of Saudi Arabia. On April 18, results of a physical examination (including chest radiograph) for a visa extension in Qatar were unremarkable. On the day of arrival back in Tunisia (April 28), the patient experienced chills, followed by arthralgia, dry cough, and fever. The daughter reported that her father had had no direct contact with camels during his stay in Qatar or Saudi Arabia. One of his children (patient 3, a nurse) gave him acetaminophen and aspirin for 3 days and then intravenously administered dexamethasone (4 mg) twice a day for 2 days. On May 6, patient 1 experienced worsened dyspnea and he sought care at the Centre Hospitalier-Universitaire Fattouma Bourguiba Hospital (Monastir, Tunisia) emergency department, where he received a fifth injection of dexamethasone. Chest radiograph showed left lower lobe infiltrate (Technical Appendix Figure). The patient was first admitted to the pulmonary ward, where he received amoxicillin-clavulanate (1 g) 3 times daily; however, on May 8, respiratory failure and peripheral signs of shock necessitated admission to the intensive care unit (ICU), where he was positioned prone and given noradrenalin infusion and mechanical ventilation with additional nitric oxide.

Mini (<10 mL fluid injected) bronchoalveolar lavage recovered a liquid of low cellularity; cultures for bacteria and fungi were negative. Serologic tests for common respiratory viruses were negative. The patient was first given amoxicillin-clavulanate, ciprofloxacin, and rifampin. On his second day in ICU, oseltamivir was added. The lavage fluid was then tested in the Tunisia National Reference Laboratory (TNRL) for MERS-CoV by using real-time reverse transcription PCR (rRT-PCR) upE (region upstream of the E gene), open reading frame (ORF) 1a, and ORF1b assays. These assays were developed in house according to the Corman et al. protocol (*3*); results were negative. On May 10, patient 1 died of multiple organ failure. Because nasopharyngeal swab samples from his 2 adult children were positive for MERS-CoV, the case of patient 1 was reported to the World Health Organization as probable MERS-CoV infection (*4*).

**Figure 2:**
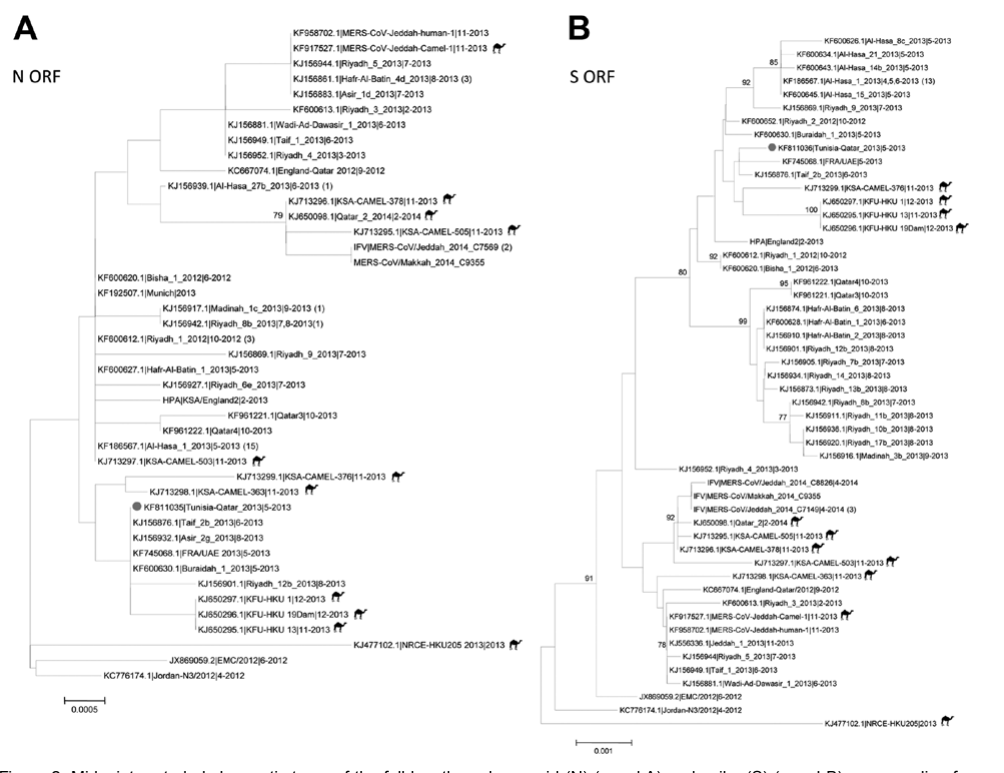
Midpoint-rooted phylogenetic trees of the full-length nucleocapsid (N) (panel A) and spike (S) (panel B) open-reading frames (ORFs) of isolates obtained from index case-patient with Middle East respiratory syndrome coronavirus (MERS-CoV) infection, Tunisia, 2013. Serum and available nucleotide sequences from GenBank and Public Health England (http://www.hpa.org.uk/webw/HPAweb&HPAwebStandard/HPAweb_C/1317136246479) and the Institut Für Virologie (http://www.virology-bonn.de/index.php?id = 46). The estimated neighbor-joining trees were constructed from nucleotide alignments by using MEGA version 6.06 (http://www.megasoftware.net). Sequence names are written as GenBank accession number|virus strain name|month-year of collection. Numbers in parentheses denote the number of additional sequences from viruses isolated from humans that are identical to the listed sequence. Solid circles indicate sequences from MERS-CoV from Tunisia, 2013. Camel icons indicate MERS-CoV sequences derived from isolates from camels. Bootstrap support values (1,000 replicates) ≥75% were plotted at the indicated internal branch nodes. Scale bars indicate number of nucleotide substitutions per site.

On August 5, 2013, the Centers for Disease Control and Prevention (CDC) tested a serum sample collected from the index-patient on May 9. Independent rRT-PCRs were positive for MERS-CoV (*5*); targets were upE (cycle threshold [C_t_] 30.27) and nucleocapsid protein (N)2 (C_t_ 30.46). Sequences of the full N and spike (S) protein coding regions were submitted to GenBank (accession nos. KF811035 and KF811036, respectively). Nucleotide/predicted amino acid sequence identities with published MERS-CoV sequences for the N and S gene coding regions ranged from 99.2%–100% to 99.0%–100% and from 99.4%–99.9% to 99.4%–99.8%, respectively. Phylogenetic relationships between this virus (designated *Tunisia-Qatar_2013*) and other published MERS-CoV sequences showed clustering with geographically diverse sequences from Saudi Arabia and the United Arab Emirates (Figure 2). 

Patient 2 was the 30-year-old daughter who had accompanied the index case-patient to Mecca. She remained in Qatar until she attended her father’s funeral in Tunisia on May 11, 2013, when she reported sore throat, cough, and fever. On May 13, a chest radiograph showed bronchial thickening. A nasopharyngeal swab sample collected on May 16 was positive for MERS-CoV by rRT-PCR performed at the TNRL: upE C_t_ 27.5, ORF1a C_t_ 27.46, and ORF1b C_t_ 37.55. Testing at CDC detected a C_t_ of 28.46 for upE and negative results for N2 and N3 (*5*). A few days after she received oseltamivir, the patient’s symptoms resolved.

Patient 3 was the 34-year-old son of the index case-patient, a nurse in the ICU where his father had been admitted. He had not traveled outside the country during the incubation period, and his first contact with the index case-patient was after his father’s return to Tunisia and illness onset. He cared for his father at home during the initial phase of illness and thereafter in the pulmonology department and ICU. Patient 3 reported a sore throat on the day after his father’s funeral. A nasopharyngeal swab sample obtained on May 16 was positive for MERS-CoV by rRT-PCR performed at TNRL: upE C_t_ 21.56, ORF1a C_t_ 27.6, and ORF1b C_t_ 31.39. At CDC, the nasopharyngeal swab sample was positive for MERS-CoV by 3 independent rRT-PCRs (*5*): C_t_ 21.67 for upE, 34.51 for N2, and 32.32 for N3. Patient 3 recovered without treatment.

Contact tracing involved the 4 remaining family members. Nasopharyngeal and/or throat swab samples were collected a mean of 5 weeks after contact from the other 2 (not ill) children of patient 1, his spouse, and the spouse of patient 3. Health care workers who had been in contact with the index case-patient in the pulmonology ward (n = 2) or ICU (n = 6) and who had reported sore throat, hyperthermia, and/or diarrhea (1 worker) were also investigated. All respiratory samples from contacts were negative for MERS-CoV by rRT-PCR.

## Conclusions

The fact that the diagnosis for the index case-patient was made by PCR of a serum sample collected 10 days after symptom onset and tested several weeks later highlights the value of testing serum samples for MERS-CoV RNA. This finding also provides valuable information about viremia in MERS CoV–infected patients, contributing to our understanding of the natural history of MERS-CoV infection and kinetics of virus shedding (*7*).

Given the incubation period of the disease (up to 15 days), the father most likely acquired his infection in Qatar (*8*,*9*). Patient 3, who had not traveled outside Tunisia, could have been exposed during the 11 days he cared for his father at home and in the hospital. The history of patient 2 is less clear; she might have acquired the virus from the same source as her father in Qatar, or she might have been secondarily infected by contact with him before he left Qatar, given that her illness began almost 12 days after her father’s.

Patient 1 was severely ill at the time of ICU admission; in <3 days, his condition rapidly evolved to multiple organ system failure and death. Although we cannot account for the diabetes or corticosteroid contributions to his disease severity, we can speculate that they might have worsened his outcome. Other MERS CoV patients who have died had concurrent conditions (*2*), and corticosteroids are thought to worsen the outcomes for patients with influenza A(H1N1) virus infection (*10*)**.**

The contact tracing results shed light on the potential for person-to-person transmissibility of MERS-CoV. Only 2 family members who had been in close and prolonged contact with the index case-patient became infected. Infection was not acquired by the case-patient’s wife, his 2 children who did not live with him, or the ICU workers who had short-term close contact with him. However, these results should be interpreted cautiously because only nasopharyngeal swab samples obtained 5 weeks after contact with the index case-patient were tested. In addition, serologic testing, which was not performed in the present investigation, could have shed more light on person-to-person MERS-CoV transmissibility.

Technical Appendix Chest radiograph of the Middle East respiratory syndrome index case-patient, taken at the time of admission to intensive care unit (May 8, 2013), Tunisia.

## References

[1] World Health Organization. Middle East respiratory syndrome coronavirus (MERS-CoV)—update. 2014 [cited 2014 May 20]. http://www.who.int/csr/don/2014_05_15_mers/en/

[2] The Who MERS-Cov Research Group. State of knowledge and data gaps of Middle East respiratory syndrome coronavirus (MERS-CoV) in humans. PLOS Currents Outbreaks. 2013:5. pii: ecurrents.outbreaks.0bf719e352e7478f8ad85fa30127ddb8 **PMID: 24270606** 10.1371/currents.outbreaks.0bf719e352e7478f8ad85fa30127ddb8 PMC3828229 24270606

[3] Corman VM, Eckerle I, Bleicker T, Zaki A, Landt O, Eschbach-Bludau M, Detection of a novel human coronavirus by real-time reverse-transcription polymerase chain reaction. Euro Surveill. 2012;17. pii: 20285. **PMID: 23041020** 10.2807/ese.17.39.20285-en 23041020

[4] World Health Organization. MERS-CoV summary and literature update—as of 31 May 2013 [cited 2013 Nov 16]. http://www.who.int/csr/disease/coronavirus_infections/update_20130531/en/

[5] Lu X, Whitaker B, Sakthivel SK, Kamili S, Rose LE, Lowe L, Real-time reverse transcription-PCR assay panel for Middle East respiratory syndrome coronavirus. J Clin Microbiol. 2014;52:67–75. 10.1128/JCM.02533-13 24153118 PMC3911421

[6] Guery B, Poissy J, el Mansouf L, Sejourne C, Ettahar N, Lemaire X, Clinical features and viral diagnosis of two cases of infection with Middle East respiratory syndrome coronavirus: a report of nosocomial transmission. Lancet. 2013;381:2265–72 . 10.1016/S0140-6736(13)60982-4 23727167 PMC7159298

[7] de Sousa R, Reusken C, Koopmans M. MERS coronavirus: data gaps for laboratory preparedness. J Clin Virol. 2014;59:4–11. **PMID: 24286807 .** 10.1016/j.jcv.2013.10.030 PMC7108266 24286807

[8] Assiri A, Al-Tawfiq JA, Al-Rabeeah AA, Al-Rabiah FA, Al-Hajjar S, Al-Barrak A, Epidemiological, demographic, and clinical characteristics of 47 cases of Middle East respiratory syndrome coronavirus disease from Saudi Arabia: a descriptive study. Lancet Infect Dis. 2013;13:752–61. 10.1016/S1473-3099(13)70204-4 23891402 PMC7185445

[9] Breban R, Riou J, Fontanet A. Interhuman transmissibility of Middle East respiratory syndrome coronavirus: estimation of pandemic risk. Lancet. 2013;382:694–9. 10.1016/S0140-6736(13)61492-0 23831141 PMC7159280

[10] Brun-Buisson C, Richard JC, Mercat A, Thiébaut AC, Brochard L. Early corticosteroids in severe influenza A/H1N1 pneumonia and acute respiratory distress syndrome. Am J Respir Crit Care Med. 2011;183:1200–6 REVA-SRLF A/H1N1v 2009 Registry Group. 10.1164/rccm.201101-0135OC 21471082

