# Supplementary material for: Family Cluster of Middle East Respiratory Syndrome Coronavirus Infections, Tunisia, 2013
Source: Emerg Infect Dis. 2014 Sep;20(9):1527–30. doi: 10.3201/eid2009.140378 (PMC4178422; doi:10.3201/eid2009.140378)
Supplement: Technical Appendix — Chest radiograph of the Middle East respiratory syndrome index case-patient, taken at the time of admission to intensive care unit (May 8, 2013), Tunisia. [file 14-0378-Techapp-s1.pdf]

Article DOI: http://dx.doi.org/10.3201/eid2009.140378

Family Cluster of Middle East Respiratory Syndrome Coronavirus Infections, Tunisia, 2013

Technical Appendix

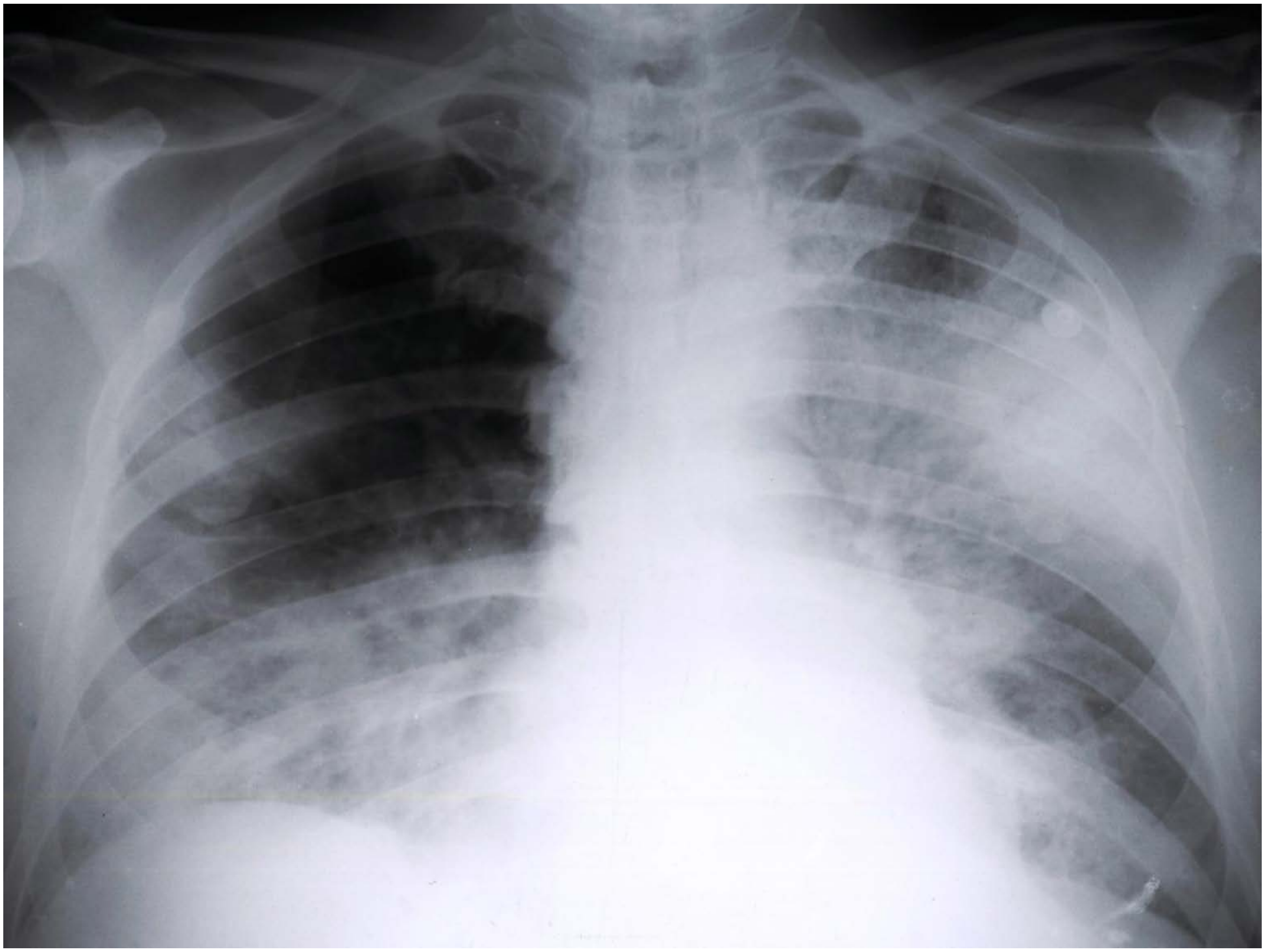

Technical Appendix Figure. Chest radiograph of the Middle East respiratory syndrome index case-patient, taken at the time of admission to intensive care unit (May 8, 2013), Tunisia, showing pulmonary infiltrates in the entire left pulmonary field and a contralateral infiltrate in the lower half of the right pulmonary field.
